# Low adherence of Swiss children to national dietary guidelines

**DOI:** 10.1016/j.pmedr.2016.03.004

**Published:** 2016-03-08

**Authors:** L. Suzanne Suggs, Sara Della Bella, Pedro Marques-Vidal

**Affiliations:** a Università della Svizzera italiana, Institute for Public Communication, Lugano, Switzerland; b Department of Internal Medicine, Lausanne University Hospital (CHUV), Lausanne, Switzerland

**Keywords:** Dietary guidelines, Eating behavior, Children, Guideline adherence, Epidemiology

## Abstract

**Introduction:**

Dietary guidelines aim to inform people of the types of foods and quantities they should consume each day or week to promote and maintain health. The aim of this study was to describe children's dietary behaviors in terms of adherence to the Swiss Society for Nutrition (SSN) dietary guidelines and possible determinants.

**Methods:**

A cross-sectional study was conducted in September 2010 with 568 children aged 6–12 years old living in Ticino Switzerland. Food intake was collected using 7-day food logs. Adherence with the dietary guidelines from the SSN was assessed according to age group.

**Results:**

With the exception of fish and cereal/potato intake (adherence rates of 68.5% and 47.9%, respectively), adherence to SSN guidelines was low: 26.9% for meat; 22.7% for eggs; 10.4% for fruit; 9.5% for sweets, snacks & soft drinks; 3.5% for milk & dairy, and 0% for vegetables. Multivariate analysis showed no consistent association between the child or their parent's socio-demographic characteristics and adherence to SSN guidelines. Girls had a higher likelihood of adhering with fruit and meat guidelines: multivariate adjusted odds ratio (95% confidence interval) 1.98 (1.10–3.56) and 1.80 (1.08–2.99), respectively. Children aged 10 to 12 had a lower likelihood of adhering with cereals and potatoes 0.48 (0.29–0.78), and a higher likelihood of adhering with the guideline for eggs 1.78 (1.00–3.15).

**Conclusion:**

Dietary intake of Ticinese children shows poor adherence with SSN guidelines. Given the lack of specific socio-demographic factors associated with adherence, population-wide interventions to improve dietary intake are necessary.

## Highlights

• Adherence to dietary guidelines of Swiss children aged 6–12 is low.
• One out of ten children adheres with fruit intake, none with vegetable intake.
• (Non)adherence rates are evenly distributed between socio-economic strata.
• 6 to 9 year old children are more likely to meet dairy and cereals guidelines.
• Overall adherence to guidelines is not significantly associated with a child's BMI.

## 1 Introduction

Unhealthy diets can lead to high blood pressure, increased blood glucose, elevated blood lipids, and obesity; all intermediate risk factors for cardiovascular diseases. Conversely, diets rich in fruits and vegetables reduce the risks of several non-communicable diseases and cancers (McNaughton et al., 2009, Fernandes et al., 2013, World Cancer Research Fund and American Institute for Cancer R, Anon., n.d; World Health Organization (WHO), 2011). Moreover, there is some evidence of linkages between cognitive functioning, academic performance and children's food intake: excessive intake of saturated fats and simple carbohydrates is adversely associated with several learning and memory processes (Correa-Burrows et al., 2015, Nyaradi et al., 2014, Northstone et al., 2012).

Despite the evidence on the importance of a healthy diet and the consequences of an unhealthy one, the overall quality of diets has declined. There has been a shift from diets high in grains and vegetables to diets rich in fat and sugar and characterized by increased consumption of processed foods (Popkin and Ng, 2007, World Health Organization (WHO), 2007). Eating habits are acquired during childhood (Hu, 2008, Krebs et al., 2007, Birch, 1999, Anzman et al., 2010) and generally endure over one's life (World Health Organization (WHO), 2014). Thus, it is critical that children follow a healthy diet.

Both the Food and Agricultural Organization and the World Health Organization have provided recommendations on energy proteins and nutrient requirement since the 1950s (European Food Safety Authority, 2007). On the contrary, food based dietary guidelines aim to inform people of the types of foods and quantities they should consume, daily and weekly, to promote and maintain health. In Switzerland, the Swiss Society for Nutrition (SSN) has issued food based guidelines for adults and children (Walter et al., 2007). Several studies have shown that adherence with the guidelines is low among Swiss adults (de Abreu et al., 2013, de Abreu et al., 2014, Meier et al., 2010). However, to date, no study has assessed dietary guideline adherence in children.

The aim of this study was to describe children's dietary behaviors in terms of adherence to the SSN 2010 dietary guidelines. Data from a sample of children living in the Italian-speaking canton of Ticino (a heterogeneous state with mountain villages, rural areas and small cities that hosts around 350,000 residents) that were attending public elementary school or first two classes of middle school and who enrolled in the project FAN (Famiglia, Attività fisica, Nutrizione) in 2010 (Suggs et al., 2013) were used.

## 2 Methods

### 2.1 Sampling

Data come from the project FAN (Famiglia, Attività fisica, Nutrizione) conducted in September 2010. Details of the project FAN have been described previously (Suggs et al., 2013). Briefly, FAN was an 8-week social marketing program offered free of charge to families in Ticino and promoted through schools. It encouraged and advised children and their parents to eat healthy and get regular physical activity. The data used in this study were collected prior to exposure to the intervention.

### 2.2 Non-dietary data collection

Through a survey administered to parents of participating children, parents were asked to provide their highest educational degree and their child's height and weight. The child's body mass index (BMI) was calculated and the child was categorized as underweight, healthy weight, overweight and obese according to age and gender-specific criteria from the U.S. Centers for Disease Control and Prevention, that have been previously validated with Swiss children aged 6–12 (Zimmermann et al., 2004).

### 2.3 Dietary data collection and adherence with dietary guidelines

Food intake of the children was collected using a paper based 7-day food log validated for this population (*insert reference after peer-review*). For each day of the week, children were asked to write down the foods and beverages they consumed at the main meals (breakfast, lunch and dinner) and between them (morning, afternoon and after dinner). Information about portion size was not collected because of known difficulties in accurate self-report of portion sizes (Livingstone and Robson, 2000, Foster et al., 2009, Collins et al., 2010, Thiagarajah et al., 2008). The 7-day food log was used for several reasons. First, there is no food frequency questionnaire for children available in Ticino. Second, daily logging of food consumption is more accurate than 3–7 day recall reporting. Finally, the instrument was tested with parents and children independently recording what the child ate each day and a high level agreement between parents and children was found (note: data are available upon request).

Coding procedures of the food log was developed in collaboration with a registered dietician and the foods were categorized into fruit; vegetables; milk, probiotics & dairy; sweets, snacks and soft drinks (SSD); cereal & potatoes; eggs; fish; and meat. An example of the foods included in each category is provided in Supplementary Table 1. As dietary guidelines include daily and weekly consumption recommendations, daily consumptions were averaged throughout the week by summing the frequency of consumption of each of the eight food categories from every meal throughout the week and dividing this count by the number of days the child completed the log), while weekly consumptions were obtained by summing the frequency of consumption of each of the eight food categories from every meal. The 2010 SSN recommendations for children are summarized in Supplementary Table 2.

A child was categorized as adhering to the SSN dietary guidelines if their consumption was within the range proposed by SSN for food consumed daily. For instance, according to the SSN guidelines, a child aged 6 should eat three portions of dairy daily. For this analysis, a 6-year old child was considered as adherent to the dairy recommendations if they ate dairy between 2.8 and 3.2 times per day. Fish and fruit over-consumers (eating fish more than once a week or eating fruits more than twice a day) were considered to be adherers, as an overconsumption of these foods was not considered harmful.

### 2.4 Statistical analyses

Statistical analyses were performed using Stata 13.0 (Stata Corp, College Station, TX, USA). Descriptive results were presented as mean ± standard deviation or as the percent or actual number of participants. Bivariate associations between adherence and several characteristics of the sample were assessed by chi-square or by Fisher's exact test. Multivariable associations were assessed by logistic regression and the results were expressed as odds ratio and (95% confidence interval). Sensitivity analyses were conducted including all data available in the bivariate analysis. All tests were two-sided and statistical significance was assessed for p < 0.05.

### 2.5 Ethics statement

Study procedures were reviewed by the Canton Ticino Ethics Committee and voted “except” from human research ethics approval in accordance with Swiss law. The recommendations in the Helsinki Declaration were followed and all participants, both children and parents, provided informed consent and voluntarily provided their data.

## 3 Results

### 3.1 Characteristics of the participants

Out of the 750 children who enrolled in FAN, 607 children completed the food log in September 2010 and 568 of them (93.6%) had complete data for analysis. Comparison between children with and without complete data showed no major differences, except that children with incomplete data were more often from a non-Swiss family (i.e. from parents without Swiss citizenship) than children with complete data (Supplemental Table 3).

Of the 568 children included, 287 (50.5%) were female and mean age was 8.5 ± 1.9 years. The frequencies of underweight, healthy weight, and overweight/obesity were 10.4%, 72.0% and 17.6%, respectively. The reported parent's education was 26.4% high, 15.5% middle and 58.1% low.

### 3.2 Adherence with dietary guidelines

The overall rates of adherence with the SSN guidelines were, in descending order, 68.5% for fish; 47.9% for cereals & potatoes; 26.9% for meat; 22.7% for eggs; 10.4% for fruit; 9.5% for SSD; 3.5% for milk & dairy and 0% for vegetables (Table 1). For SSD, all non-adheres were over-consumers, a similar trend being for meat, for which 72.7% were over-consumers. Conversely, for eggs and cereals & potatoes, most non-adheres were under-consumers (71.0% and 51.6%, respectively).

### 3.3 Factors associated with adherence

The bivariate associations between adherence with the SSD guidelines and the characteristics of the children and their parents are summarized in Table 1. Children aged 10 to 12 had a lower adherence to cereal & potato recommendations and a higher adherence to egg consumption; girls had a higher adherence to fruit and meat guidelines. Children whose parents were Swiss and children whose parents were obese or with a healthy weight had a higher adherence to meat guidelines, but the numbers were rather small. Finally, both children who took part in the FAN project with their mothers and children whose parents were non-Swiss had a higher adherence to fruit guidelines (Table 1).

Most of the findings were reinforced by the multivariate analysis (Table 2a, Table 2b). Regarding the characteristics of children, increasing age was inversely associated with adherence to cereals and potatoes and milk and dairy guidelines, although the last association was borderline significant (p = 0.054); increasing age was also positively associated with adherence to egg consumption. Girls had a higher likelihood of adhering to fruit and meat guidelines. BMI was not significantly associated with the likelihood of adhering with any guidelines except the meat one: obese children seem to have an increase likelihood of adhering with meat guidelines (although this association is only marginally significant) (Table 2a). Regarding the characteristics of parents, children of non-Swiss parents had a lower likelihood of adhering with meat guidelines, but a higher likelihood of adhering with fruit guidelines. Children who took part in the project with their mothers also had a higher likelihood of adhering with fruit guidelines, while no association was found with BMI or educational level of the reporting parent (Table 2b).

### 3.4 Sensitivity analyses

The results of the sensitivity analysis using all available data are summarized in Supplementary Tables 3 and 4. Neither the overall rates of adherence nor the results of the bivariate analysis changed substantively.

## 4 Discussion

To our knowledge, this is the first study ever conducted in Switzerland assessing adherence with SSN guidelines in school-aged children. Our results demonstrate that adherence levels are low and that consumption of SSD and meat is excessive in this group. Given the importance of consuming a healthy diet in childhood, the results obtained in this study are concerning and will serve as a reference for future descriptive or intervention studies among Swiss school-aged children.

### 4.1 Adherence with dietary guidelines

Overall, the children in this study displayed low adherence with the SSN dietary guidelines. They consumed too many sweets, snacks and soft drinks and ate too few fruits and vegetables. They did not consume enough dairy and ate too much meat. All in all, they mirror the low adherence to guidelines observed among Swiss adults (de Abreu et al., 2013, Meier et al., 2010).

### 4.2 Factors associated with adherence

Girls were more likely to meet or exceed recommendations concerning fruit intake and were less likely to exceed the recommended intake of meat. These findings are in agreement with a study of children aged 6–14 in the German part of Switzerland, where boys consumed more meat and less fruit than girls (Aeberli et al., 2007). Boys and girls seem to differ in their food preferences and attitudes towards foods and cultural issues might be at play since some foods are perceived to be more masculine than others (meat vs fruit) (Cooke and Wardle, 2005, Arganini et al., 2012). Another factor that could contribute to gender differences in food choice may stem from girls' concern about weight control: some studies suggest that gender differences are apparent even among children and adolescents, with females more likely to experience higher level of body dissatisfaction and weight concerns (Arganini et al., 2012, Lowes and Tiggemann, 2003, Johnson and Wardle, 2005, Mendes et al., 2014, Furnham et al., 2002). Moreover, females have been shown to be more health conscious and possess better health-related nutrition knowledge (Baker and Wardle, 2003, Pirouznia, 2001, Wardle et al., 2000).

Younger (i.e. 6-year old) children showed a better adherence to dietary guidelines for fruits and cereals & potatoes. Conversely, older (10–12 years old) children were more likely to meet the guidelines for egg intake. Some studies suggest that “older” children (i.e. adolescents) have a less healthy diet because of lower parental control, increased peer pressures and autonomy in the choice of food (Birch et al., 2007, Gidding, 2005). However, other studies showed a non-linear association between age and food preferences (Cooke and Wardle, 2005), suggesting that among pre-school children the younger children are more affected by peer pressure than the older ones (Birch, 1980) and that younger adolescents are more susceptible to peer influence and hence tend to have a worse diet (Nørgaard et al., 2013).

No association was found between child or parent BMI status and adherence to most of the SSN dietary guidelines. The only exception is meat, for which there is a positive association between being obese and adhering to the guidelines, although this association is only marginally significant and we need to be very cautious in interpreting these findings in that reverse causality might partly account for them. These findings are not consistent with a recent study of 6 to 14-year-old children living in the German part of Switzerland that found that overweight children had a higher intake of meat and protein (Aeberli et al., 2007). Further, many studies that compared the food intake of obese and non-obese children reported mixed findings (Krebs et al., 2007, Field et al., 2003, Ludwig et al., 2001, Bradlee et al., 2010, Lahti-Koski et al., 2002). Although a reporting bias cannot be ruled out (i.e. overweight parents or children voluntarily underestimating their meat intake), our results fail to find a consistent association between BMI status and adherence to dietary guidelines.

Many studies have shown that children from lower SES families show less healthy eating behaviors such as skipping breakfast, consuming less fruits and vegetables and more sugar sweetened drinks (Fernández-Alvira et al., 2013, Ahmadi et al., 2015, Adamo and Brett, 2014, Xie et al., 2003). Conversely, higher parental education has been shown to be associated with healthier food choices, higher vegetable consumption and lower intake of sugars and fats (Fernández-Alvira et al., 2013, Ahmadi et al., 2015, Adamo and Brett, 2014, Xie et al., 2003). In this study, no association was found between the education level of the parent and their child's adherence to dietary guidelines. Possible explanations are that parents are unaware of the guidelines regarding healthy eating or tend to be less strict regarding their child's eating behavior. Overall, our results suggest that, in Ticino, the parent's education level is not associated with a healthier dietary intake in children, but further studies are needed to assess the reasons for this absence of association.

Children of migrant parents had a higher likelihood of adhering to guidelines for fruit intake, a finding in agreement with other studies conducted among adults (de Abreu et al., 2013). This finding is likely related to the fact that the majority of migrants in Ticino are of Italian origin, a country with higher fruit consumption per capita than Switzerland (Abreu et al., 2014).

### 4.3 Importance for public health and prevention efforts

Healthy eating behaviors should be promoted and established during childhood. Our results indicate that children in Ticino consume too many sweets, snacks and soft drinks, too much meat and eat too few fruits and vegetables. Thus, appropriate interventions are needed to curb impending health problems associated with unhealthy eating behaviors.

Many approaches have been implemented around the globe, some with success and others with marginal to no effect. Many interventions target overweight and/or obese children, running the risk of stigmatizing children with “weight problems” thereby missing the opportunity to reinforce good behaviors or focus on a population based norm or healthy eating. Promoting healthy diets requires a combination of interventions at the individual as well as the environmental level. Interventions relying on communication and education are less effective than interventions using more holistic approaches reflecting the social and environmental influences on individual lifestyle choices (Nutbeam, 2000, Merzel and D'Afflitti, 2003). For example, school meal interventions would be of little utility in Ticino, because most children return home for lunch. Social marketing has been shown to effectively intervene at individual, community, environmental and policy levels (Stead et al., 2007a, Stead et al., 2007b, Evans et al., 2010, Wolfenden et al., 2014, Gordon et al., 2006) and as such, a social marketing approach to dietary improvement is warranted.

### 4.4 Strengths and limitations

This study is characterized by several strengths. This is the first study in Switzerland to assess school-aged children's eating behaviors and their adherence with national dietary guidelines. The sample well resembles Ticino's population and includes children from all BMI categories. It examined children's food and beverage intake using a 7-day log. Food consumption logs have several advantages compared to other dietary assessment methods, including food frequency questionnaires and 24 h-recall, as they are completed in an ongoing basis, not relying on children's ability to recall what they ate 1 day ago, or select only from a list of items. They also provide a better estimate of typical weekly food intake which allows us to overcome day to day variability in eating behaviors (Livingstone and Robson, 2000, Collins et al., 2010, Bingham et al., 1994). Further, they allow assessing adherence to guidelines based on weekly consumptions, something unachievable by 24 h-recalls.

This study also has some limitations. First, it relied on voluntary participation in the FAN project, and it is known that participants in voluntary health related studies tend to be more health conscious than the general population. Hence, it is possible that our results might be biased towards a more positive dietary behavior, and that the true adherence to SSN guidelines in the entire population might be even lower. However, this would place even more urgency in the need to promote healthy eating behaviors among Ticinese children, given the low adherence with guidelines found in our sample. Second, as with all studies using self-reported data, self-reporting errors likely exist. However, this cannot be resolved without more invasive data collection measures, such as direct or video observation or biometric samples. Seasonal variation in food intake could not be accounted for (Ma et al., 2005, Shahar et al., 2001, Rossato et al., 2010) and it is not known if children's adherence to the SSN dietary guidelines varies over the year; although it is unlikely that seasonal variation would considerably change adherence rates. Finally, we acknowledge that the cross-sectional nature of our data limited our analysis and interpretation in that we could not control for potential reverse causation.

## 5 Conclusion

Children aged 6–12 living in Ticino have a low adherence to the SSN dietary guidelines. Population-based interventions are needed to improve the dietary consumption behavior of Swiss children.

## Conflict of interest statement

The authors report no conflict of interest.

## Funding

This study was funded by the Swiss National Science Foundation (Ref. n. CR13I3_156385/1). The FAN intervention was funded by the Department of Health and Social Services of Canton Ticino and by Health Promotion Switzerland. The funders had no role in the decision to publish or in the preparation of the manuscript.

## Transparency document

Transparency document. Image 1

## Figures and Tables

**Table 1:** Bivariate analysis of the factors associated with children's adherence to the guidelines of the Swiss society of Nutrition, canton Ticino, 2010, N = 568.

	N	Fruit	Milk & dairy	Cereals & potatoes	SSD	Eggs	Fish	Meat
All	568	59 (10.4)	20 (3.5)	272 (47.9)	54 (9.5)	129 (22.7)	389 (68.5)	153 (26.9)
Child's data								
Age group (years)								
6	116	18 (15.5)	7 (6.0)	66 (56.9)	8 (6.9)	23 (19.8)	79 (68.1)	32 (27.6)
7 to 9	271	25 (9.2)	10 (3.7)	135 (49.8)	26 (9.6)	53 (19.6)	185 (68.3)	76 (28.0)
10 to 12	181	16 (8.8)	3 (1.7)	71 (39.2)	20 (11.1)	53 (29.3)	125 (69.1)	45 (24.9)
p-Value		0.126	0.123 §	0.008	0.491	0.038	0.979	0.745
Gender								
Male	281	21 (7.5)	11 (3.9)	139 (49.5)	24 (8.5)	62 (22.1)	188 (66.9)	64 (22.8)
Female	287	38 (13.2)	9 (3.1)	133 (46.3)	30 (10.5)	67 (23.3)	201 (70.0)	89 (31)
p-Value		0.024	0.655 §	0.456	0.437	0.716	0.422	0.027
BMI categories								
Underweight	59	8 (13.6)	1 (1.7)	30 (50.9)	3 (5.1)	14 (23.7)	37 (62.7)	15 (25.4)
Healthy weight	409	41 (10.0)	14 (3.4)	202 (49.4)	36 (8.8)	96 (23.5)	282 (69)	108(26.4)
Overweight	67	6 (9.0)	4 (6.0)	28 (41.8)	11 (16.4)	14 (20.9)	49 (73.1)	19 (28.4)
Obese	33	4 (12.1)	1 (3.0)	12 (36.4)	4 (12.1)	5 (15.2)	21 (63.6)	11 (33.3)
p-Value		0.763 §	0.590 §	0.343	0.138	0.713	0.576	0.830
Parent's data								
Gender								
Male	77	3 (3.9)	3 (3.9)	39 (50.7)	10 (13.0)	17 (22.1)	46 (59.7)	15 (19.5)
Female	491	56 (11.4)	17 (3.5)	233 (47.5)	44 (9.0)	112 (22.8)	343 (69.9)	138 (28.1)
p-Value		0.045 §	0.744 §	0.602	0.263	0.887	0.076	0.113
BMI categories								
Underweight	34	4 (11.8)	1 (2.9)	20 (58.8)	2 (5.9)	7 (20.6)	25 (73.5)	7 (20.5)
Healthy weight	364	39 (10.7)	12 (3.3)	169 (46.4)	29 (8.0)	80 (22.0)	250 (68.7)	114 (31.3)
Overweight	129	16 (12.4)	5 (3.9)	63 (48.8)	17 (13.2)	35 (27.1)	91 (70.5)	21 (16.3)
Obese	41	0 (0.0)	2 (4.9)	20 (48.8)	6 (14.6)	7 (17.1)	23 (56.1)	11 (26.8)
p-Value		0.071 §	0.882 §	0.572	0.181 §	0.498	0.311	0.007 §
Parent's education								
Primary	19	1 (5.3)	1 (5.3)	7 (36.8)	2 (10.5)	2 (10.5)	12 (63.2)	2 (10.5)
Secondary	356	33 (9.3)	11 (3.1)	166 (46.6)	32 (9.0)	83 (23.3)	250 (70.2)	102 (28.6)
Tertiary	193	25 (13.0)	8 (4.2)	99 (51.3)	20 (10.4)	44 (22.8)	127 (65.8)	49(25.4)
p-Value		0.382 §	0.481 §	0.378	0.762 §	0.431	0.499	0.192 §
Nationality								
Non-Swiss	66	15 (22.7)	5 (7.6)	31 (47.0)	7 (10.6)	9 (13.6)	44 (66.7)	10 (15.2)
Swiss	502	44 (8.8)	15 (3.0)	241 (48.0)	47 (9.4)	120 (23.9)	345 (68.7)	143 (28.5)
p-Value		< 0.001	0.07	0.874	0.746	0.061	0.735	0.022

BMI, body mass index; SSDs, sweets, snacks and soft drinks. Results are expressed as number of participants and (row percentage) of adherers with each food group guideline. Statistical analysis by chi-square or Fisher's exact test (§). No results shown for vegetables as no child in our sample adhered with the guidelines concerning vegetables' intake.

**Table 2a:** Multivariate analysis of the children-related factors associated with children's adherence to the guidelines of the Swiss Society of Nutrition, Canton Ticino, 2010, N = 568.

	Fruit	Milk & dairy	Cereals & potatoes	SSD	Eggs	Fish	Meat
Gender							
Male	1 (ref.)	1 (ref.)	1 (ref.)	1 (ref.)	1 (ref.)	1 (ref.)	1 (ref.)
Female	1.98 (1.10–3.56)	0.72 (0.29–1.81)	0.80 (0.57–1.12)	1.35 (0.75–2.40)	1.10 (0.74–1.65)	1.17 (0.81–1.68)	1.53 (1.03–2.25)
Age group (years)							
6	1 (ref.)	1 (ref.)	1 (ref.)	1 (ref.)	1 (ref.)	1 (ref.)	1 (ref.)
7 to 9	0.57 (0.29–1.13)	0.59 (0.21–1.63)	0.75 (0.48–1.17)	1.55 (0.67–3.60)	1.03 (0.59–1.79)	1.06 (0.66–1.71)	1.04 (0.63–1.72)
10 to 12	0.59 (0.28–1.26)	0.25 (0.06–1.02)	0.48 (0.29–0.78)	1.92 (0.8–4.61)	1.78 (1.00–3.15)	1.08 (0.65–1.81)	0.87 (0.50–1.51)
p-Value for trend	0.172	0.053	0.003	0.147	0.049	0.764	0.624
BMI categories							
Underweight	1.64 (0.69–3.91)	0.49 (0.06–3.92)	1.02 (0.58–1.78)	0.63 (0.18–2.15)	1.15 (0.60–2.23)	0.74 (0.41–1.32)	0.87 (0.45–1.65)
Healthy weight	1 (ref.)	1 (ref.)	1 (ref.)	1 (ref.)	1 (ref.)	1 (ref.)	1 (ref.)
Overweight	0.95 (0.37–2.48)	1.58 (0.48–5.16)	0.70 (0.41–1.20)	1.90 (0.89–4.07)	0.91 (0.48–1.76)	1.34 (0.74–2.44)	1.32 (0.72–2.43)
Obese	1.17 (0.37–3.78)	0.72 (0.09–5.86)	0.52 (0.25–1.11)	1.30 (0.42–4.02)	0.58 (0.21–1.59)	0.86 (0.40–1.83)	1.96 (0.88–4.37)
p-Value for trend	0.975	0.722	0.096	0.258	0.239	0.606	0.064

BMI, body mass index; SSDs, sweets, snacks and soft drinks; NA; not assessed. For each food group guideline, results are expressed as odds ratio and (95% confidence interval). Statistical analysis by logistic regression adjusting for all variables in the table. Analysis not performed on vegetables, as no child adhered with the guidelines concerning vegetables.

**Table 2b:** Multivariate analysis of the parent's-related factors associated with children's adherence to the guidelines of the Swiss Society of Nutrition, Canton Ticino, 2010, N = 568.

	Fruit	Milk & dairy	Cereals & potatoes	SSD	Eggs	Fish	Meat
Gender							
Male	1 (ref.)	1 (ref.)	1 (ref.)	1 (ref.)	1 (ref.)	1 (ref.)	1 (ref.)
Female	3.85 (1.10–13.4)	0.86 (0.22–3.32)	0.94 (0.56–1.57)	0.82 (0.37–1.82)	1.23 (0.66–2.29)	1.57 (0.92–2.67)	1.35 (0.71–2.58)
BMI categories							
Underweight	1.05 (0.33–3.30)	0.88 (0.11–7.29)	1.49 (0.71–3.11)	0.87 (0.20–3.90)	0.93 (0.38–2.25)	1.33 (0.60–2.99)	0.59 (0.25–1.43)
Healthy weight	1 (ref.)	1 (ref.)	1 (ref.)	1 (ref.)	1 (ref.)	1 (ref.)	1 (ref.)
Overweight	1.55 (0.78–3.08)	0.99 (0.32–3.09)	1.13 (0.73–1.74)	1.63 (0.82–3.25)	1.51 (0.91–2.49)	1.15 (0.72–1.85)	0.40 (0.23–0.70)
Obese	NA	1.25 (0.25–6.34)	1.26 (0.64–2.48)	1.73 (0.63–4.73)	0.90 (0.37–2.19)	0.57 (0.28–1.12)	0.80 (0.37–1.07)
p-Value for trend	NA	0.798	0.794	0.349	0.858	0.129	0.416
Education							
Primary	1 (ref.)	1 (ref.)	1 (ref.)	1 (ref.)	1 (ref.)	1 (ref.)	1 (ref.)
Secondary	1.77 (0.20–15.6)	0.74 (0.08–6.50)	1.55 (0.58–4.12)	0.85 (0.18–4.02)	2.34 (0.52–10.5)	1.37 (0.51–3.66)	3.77 (0.48–29.6)
Tertiary	2.30 (0.26–20.6)	0.87 (0.09–8.18)	1.85 (0.68–5.07)	1.08 (0.22–5.30)	2.57 (0.56–11.8)	1.14 (0.41–3.12)	3.09 (0.38–24.8)
p-Value for trend	0.455	0.900	0.230	0.927	0.225	0.806	0.147
Nationality							
Swiss	1 (ref.)	1 (ref.)	1 (ref.)	1 (ref.)	1 (ref.)	1 (ref.)	1 (ref.)
Non-Swiss	3.44 (1.71–6.92)	2.60 (0.88–7.67)	0.98 (0.58–1.66)	1.04 (0.44–2.44)	0.50 (0.24–1.05)	0.94 (0.54–1.64)	0.43 (0.21–0.87)

BMI, body mass index; SSDs, sweets, snacks and soft drinks; NA; not assessed. For each food group guideline, results are expressed as odds ratio and (95% confidence interval). Statistical analysis by logistic regression adjusting for all variables in the table. NA not assessable. Analysis not performed on vegetables as no child adhered with the guidelines concerning vegetables.

## Appendix A Supplementary data

Supplementary tables: Table 1 Food categories; Table 2 The SSN 2010 guidelines: Tables 3 and 4 Results of sensitivity analysis. Image 1
