# Supplementary material for: Low adherence of Swiss children to national dietary guidelines
Source: Prev Med Rep. 2016 Mar 8;3:244–9. doi: 10.1016/j.pmedr.2016.03.004 (PMC4929232; doi:10.1016/j.pmedr.2016.03.004)
Supplement: Supplementary file 2 — Supplementary tables: Table 1 Food categories; Table 2 The SSN 2010 guidelines: Tables 3 and 4 Results of sensitivity analysis. [file mmc2.pdf]

Supplementary tables

Supplementary table 1. Food categories

**Food category**	**Items**
Fruit	fresh fruits, dried fruits, baked fruits and 100% fruit juices
Vegetables	fresh and cooked vegetables, 100% vegetable juice, vegetable soups, salads and mushrooms.
Milk and Dairy	milk, yogurt, several kind of cheese, white sauce, probiotic drinks
Sweets, Snacks and Soft drinks (SSD)	a) cookies, cakes, honey, etc.<br>b) chips, popcorn and salted snacks.<br>c) sweetened fruit juice, iced tea in a can/bottle, carbonated sugary drinks
Cereals and Potatoes	refined and whole grain cereals and potatoes
Eggs	eggs and eggs used to prepare meals (such as spätzli, crepes, flan, pancake, quiche, tortilla with potatoes)
Fish	fresh and canned fish as well as sea fruits
Meat	red and white meat, cold cuts and salami

Supplementary table 2. Swiss Society of Nutrition, Guidelines for children (2010).

Food	Age 4 – 6 years		Age 7 – 9 years		Age 10 – 12 years	
	**Portion number**	**Portion size**	**Portion number**	**Portion size**	**Portion number**	**Portion size**
Daily						
Fruit	2	1 portion=100gr	2	1 portion=110gr	2	A portion=120gr
Vegetables	3	1 portion=70gr	3	1 portion=70gr	3	A portion=80gr
Milk and dairy	3	1 portion=1 dl of milk or 100gr of yogurt or 100gr fresh cheese (ricotta) or 15gr hard cheese or 30gr soft cheese	2-3	1 portion=2 dl of milk or 180gr of yogurt or 200gr fresh cheese or 30gr hard cheese or 60gr soft cheese	3	1 portion=2 dl of milk/180gr of yogurt 200gr fresh cheese /30 g hard cheese and 60 g soft cheese
Sweets, snacks and soft drinks (SSD)	Max.1	1 portion=a little slice of cake or 3 biscuits/cookies *petit beurre* or a (2dl) glass of a soft drink like iced tea or cola	Max.1	1 portion=a little slice of cake or 3 biscuits/cookies *petit beurre* or a (2dl) glass of a soft drink like iced tea or cola	Max.1	1 portion=a little slice of cake or 3 biscuits/cookies *petit beurre* or a (2dl) glass of a soft drink like iced tea or cola
Cereals, potatoes	3/4	1 portion of bread=50gr 1 portion of (raw) rise or pasta or similar=40gr 1 portion of potatoes=180gr.	3-4	1 portion of bread=65gr 1 portion of (raw) rice or pasta or similar=45gr 1 portion of potatoes=220gr.	3-4	1 portion of bread=100gr 1 portion of (raw) rice or pasta or similar=60gr 1 portion of potatoes=270gr
Weekly						
Eggs	2	Included those used to cook pies or custard etc.	2	Included those used to cook pies or custard etc.	2-3	Included those used to cook pies or custard etc.
Fish	1	1 portion=50 g	1	1 portion=75gr	1	1 portion=90gr
Meat	Max.5	1 portion=50gr (cold cuts or sausages maximum once a week)	Max.5	A portion=70gr (cold cuts or sausages maximum once a week)	Max.5	A portion=85gr (cold cuts or sausages maximum once a week)

Dietary guidelines (in Italian, French or German) can be downloaded from http://www.sge-ssn.ch/it/io-e-te/mangiare-e-bere/giovani-e-anziani/bambini/

Supplemental table 3: Comparison between children with and without complete information, N=607.

	Complete (N=568)	Incomplete (N=39)	P-value
Children characteristics			
Age (years)	8.5 ± 1.9	8.5 ± 1.8	0.933
Girls (%)	287 (50.5)	20 (51.3)	0.927
Body mass index (kg/m^2)	16.5 ± 2.6	16.1 ± 2.4	0.546
Reporting parent characteristics			
Age (years)	41.1 ± 4.7	40.3 ± 4.4	0.390
Women (%)	491 (86.4)	34 (87.2)	0.897
Body mass index (kg/m^2)	23.5 ± 4.2	22.6 ± 4.1	0.285
Swiss nationality (%)	502 (88.4)	21 (67.7)	0.001
Education (%)			0.134 §
Primary	19 (3.3)	2 (12.5)	
Secondary	356 (62.7)	9 (56.3)	
Tertiary	193 (34.0)	7 (31.3)	

Results are expressed as number of children (column percentage) or as mean ± standard deviation. Statistical analysis using chi-square or Fisher's exact test (§) for qualitative variables or Student's t-test for quantitative variables.

Supplementary table 4. Bivariate analysis of the factors associated with children's adherence to the guidelines of the Swiss society of Nutrition, Canton Ticino, 2010, all children with available data, N=607.

	Fruit	Milk & dairy	Cereals & potatoes	SSD	Eggs	Fish	Meat
All	62 (10.2)	22 (3.6)	286 (47.1)	60 (9.9)	136 (22.4)	414 (68.2)	167 (27.5)
**Child's data**							
Age group (years)							
6	18 (14.8)	8 (6.6)	68 (55.7)	9 (7.4)	25 (20.5)	85 (69.7)	33 (27.1)
7 to 9	26 (8.9)	11 (3.8)	143 (49.0)	30 (10.3)	56 (19.2)	196 (67.1)	85 (29.1)
10 to 12	18 (9.3)	3 (1.6)	75 (38.9)	21 (10.9)	55 (28.5)	133 (68.9)	49(25.4)
p-value	0.178	0.066	0.009	0.570	0.047	0.851	0.663
Gender							
Male	22 (7.3)	12 (4.0)	144 (48.0)	27 (9.0)	67 (22.3)	200 (66.7)	72 (24.0)
Female	40 (13.0)	10 (3.3)	142 (46.3)	33 (10.8)	69 (22.5)	214 (69.7)	51 (16.6)
p-value	0.021	0.625	0.667	0.470	0.966	0.421	0.055
BMI categories							
Underweight	8 (13.1)	1 (1.6)	31 (50.8)	3 (4.9)	14 (23.0)	38 (62.3)	15 (24.6)
Healthy weight	42 (9.9)	14 (3.3)	207 (48.9)	38 (9.0)	100 (23.6)	291 (68.8)	114 (26.9)
Overweight	6 (8.6)	4 (5.7)	28 (40.0)	11 (15.7)	15 (21.4)	51 (72.9)	19 (27.1)
Obese	4 (12.1)	1 (3.0)	12 (36.4)	4 (12.1)	5 (15.2)	21 (63.6)	11 33.3)
p-value	0.762 §	0.615	0.280	0.172 §	0.719	0.559	0.838
**Parent's data**							
Gender							
Male	3 (3.7)	3 (3.7)	40 (48.8)	11 (13.4)	19 (23.2)	49 (59.8)	15 (18.3)
Female	59 (11.2)	19 (3.6)	246 (46.9)	49 (9.3)	117 (22.3)	365 (69.5)	152 (28.9)
p-value	0.031 §	1.000 §	0.746	0.249	0.858	0.077	0.044
BMI categories							
Underweight	4 (11.4)	1 (2.9)	20 (57.1)	3 (8.6)	7 (20.0)	25 (71.4)	8 (22.8)
Healthy weight	40 (10.4)	14 (3.7)	178 (46.4)	32 (8.3)	82 (21.4)	263 (68.5)	120 (31.3)
Overweight	16 (12)	5 (3.8)	64 (48.1)	18 (13.5)	35 (26.3)	94 (70.7)	23 (17.3)
Obese	0 (0.0)	2 (4.8)	20 (47.6)	6 (14.3)	7 (16.7)	23 (54.8)	12 (28.6)
p-value	0.073 §	0.931 §	0.676	0.232 §	0.511	0.256	0.015 §
Parent's education							
Primary	2 (9.5)	2 (9.5)	7 (33.3)	2 (9.5)	2 (9.5)	13 (61.9)	2 (9.5)
Secondary	33 (9.0)	12 (3.3)	170 (46.6)	33 (9.0)	85 (23.3)	257 (70.4)	108 (29.6)
Tertiary	25 (12.5)	8 (4.0)	103 (51.5)	23 (11.5)	46 (23.0)	131 (65.5)	53 (26.5.)
Other	0 (0.0)	0 (0.0)	4 (44.4)	1 (11.1)	0 (0.0)	6 (66.7)	2 (22.2.0)

p-value	0.547 §	0.385 §	0.382 §	0.684 §	0.219 §	0.558 §	0.218 §
Nationality							
Non-Swiss	16 (21.1)	6 (7.9)	34 (44.7)	9 (11.8)	9 (11.8)	52 (68.4)	12 (15.8)
Swiss	44 (8.4)	16 (3.1)	251 (48)	50 (9.6)	124 (23.7)	357 (68.3)	153 (29.3)
p-value	0.001	0.036	0.595	0.533	0.020	0.978	0.014

BMI, body mass index; SSD, sweets, snacks and soft drinks. Results are expressed as row percentage of adherers with each food group guideline. Statistical analysis by chi-square or Fisher's exact test (§). Analysis not performed on vegetables as no child adhered with guidelines concerning vegetables.
